# Editorial: The impact of online addiction on general health, wellbeing and associated societal costs, volume II

**DOI:** 10.3389/fpubh.2022.1018760

**Published:** 2022-09-21

**Authors:** Georgios Floros, Ioanna Mylona

**Affiliations:** ^1^2nd Department of Psychiatry, Aristotle University of Thessaloniki, Thessaloniki, Greece; ^2^Department of Ophthalmology, General Hospital of Katerini, Katerini, Greece

**Keywords:** Internet Addiction, online gaming addiction, social media addiction, online gambling addiction, wellbeing

Twenty-five years since the first mention of Internet Addiction by the late pioneer, Kimberly Young (1), and following considerable controversy regarding its classification and very existence (2), the tide has shifted to the grudging acceptance of at least a number of its facets. Internet Gaming Disorder (IGD) was the first technological addiction to be accepted as a valid diagnosis, mainly due to the steep increase of clinical cases (3). As such, it is a diagnosis that has been born out of clinical necessity and not constructed *via* scientific consensus, a rarity in our days, where considerable criticism has been rightly applied to a constant reclassification of mental disease without compelling clinical evidence for the necessity or benefit of the attempted changes.

The inclusion however of a single aspect of Internet Addiction is still a compromise. Numerous other forms of addictive behaviors are propagated *via* the Internet, including social media addiction, online content addiction, online pornography addiction, and online pathological gambling, with a very high degree of association one to another as has been evident for some time (4), as have been the correlates with chemical addictions (5). In line with this reality, this Research Topic has examined the most prominent forms of online addiction, Internet Gaming Disorder (IGD), Social Media addiction (SMA), but also a new addictive behavior, that of online content consumption, in the form of short-form video addiction.

Gan et al. investigated the cumulative effect of family risk factors on adolescent IGD and the serial mediating effects of personal growth initiative (PGI) and gratitude in a sample of 600 Chinese adolescents with the aid of a chain mediation model. The researched family risk factors included family structure, parental educational level, family economic situation, intimacy, and conflict. Results indicated that the cumulative total of family risk factors is associated with IGD with this association mediated *via* PGI and gratitude.

Ye et al. sought to determine the causes of short-form video addiction and its impact on the psychology of learning, and to investigate the relationship between short-form video flow experience, short-form video addiction, intrinsic and extrinsic learning motivation, and learning wellbeing from the perspectives of flow experience theory and micro ecological systems. They employed an online questionnaire survey of 517 Chinese students and concluded that addiction to short videos has a negative impact on learners' learning motivation and positive psychology of learning.

Chen et al. studied the social media use of 1,163 Taiwanese adults and concluded that the negative health effects of SMA may not be simply due to prolonged internet use. Furthermore, they uncovered an unexpected finding, that regular exercise increases the impact of SMA on internet use, although it did alleviate the negative impact of social media addiction on physical health.

Finally, Šablatúrová et al. provided a useful validation of the Social Media Disorder Scale in a large (13,377) representative sample of Czech adolescents.

We hope that the articles of this Research Topic will help to further our understanding not only of individual aspects of Internet Addiction, but most importantly, of the complex interrelationships between these aspects, and the eventual adoption of an umbrella definition for this complex phenomenon that has negatively impacted a large number of lives.

## Author contributions

All authors listed have made a substantial, direct, and intellectual contribution to the work and approved it for publication.

## Conflict of interest

The authors declare that the research was conducted in the absence of any commercial or financial relationships that could be construed as a potential conflict of interest.

## Publisher's note

All claims expressed in this article are solely those of the authors and do not necessarily represent those of their affiliated organizations, or those of the publisher, the editors and the reviewers. Any product that may be evaluated in this article, or claim that may be made by its manufacturer, is not guaranteed or endorsed by the publisher.
